# Supplementary material for: Individualised and non-contact post-mortem interval determination of human bodies using visible and thermal 3D imaging
Source: Nat Commun. 2021 Oct 14;12:5997. doi: 10.1038/s41467-021-26318-4 (PMC8517003; doi:10.1038/s41467-021-26318-4)
Supplement: Supplementary file 2 — Reporting Summary [file 41467_2021_26318_MOESM2_ESM.pdf]

nature portfolio

Corresponding author(s): Prof. Maurice Aalders

Last updated by author(s): Aug 31, 2021

Reporting Summary

Nature Portfolio wishes to improve the reproducibility of the work that we publish. This form provides structure for consistency and transparency in reporting. For further information on Nature Portfolio policies, see our Editorial Policies and the Editorial Policy Checklist.

Statistics

For all statistical analyses, confirm that the following items are present in the figure legend, table legend, main text, or Methods section.

n/a	Confirmed	
☐	☒	The exact sample size (n) for each experimental group/condition, given as a discrete number and unit of measurement
☐	☒	A statement on whether measurements were taken from distinct samples or whether the same sample was measured repeatedly
☒	☐	The statistical test(s) used AND whether they are one- or two-sided<br>*Only common tests should be described solely by name; describe more complex techniques in the Methods section.*
☒	☐	A description of all covariates tested
☒	☐	A description of any assumptions or corrections, such as tests of normality and adjustment for multiple comparisons
☐	☒	A full description of the statistical parameters including central tendency (e.g. means) or other basic estimates (e.g. regression coefficient) AND variation (e.g. standard deviation) or associated estimates of uncertainty (e.g. confidence intervals)
☒	☐	For null hypothesis testing, the test statistic (e.g. F, t, r) with confidence intervals, effect sizes, degrees of freedom and P value noted<br>*Give P values as exact values whenever suitable.*
☒	☐	For Bayesian analysis, information on the choice of priors and Markov chain Monte Carlo settings
☒	☐	For hierarchical and complex designs, identification of the appropriate level for tests and full reporting of outcomes
☒	☐	Estimates of effect sizes (e.g. Cohen's d, Pearson's r), indicating how they were calculated

Our web collection on statistics for biologists contains articles on many of the points above.

Software and code

Policy information about availability of computer code

Data collection	OneWire Viewer 1.0
Data analysis	Agisoft PhotoScan Professional 1.2, OneWire Viewer 1.0, MATLAB 2018b, FLIR Tools+ 5.13, https://doi.org/10.5281/zenodo.5070592

For manuscripts utilizing custom algorithms or software that are central to the research but not yet described in published literature, software must be made available to editors and reviewers. We strongly encourage code deposition in a community repository (e.g. GitHub). See the Nature Portfolio guidelines for submitting code & software for further information.

Data

Policy information about availability of data

All manuscripts must include a data availability statement. This statement should provide the following information, where applicable:

- Accession codes, unique identifiers, or web links for publicly available datasets
- A description of any restrictions on data availability
- For clinical datasets or third party data, please ensure that the statement adheres to our policy

The authors declare that all data supporting the findings of this study are present within the paper. The raw data are protected and are not available due to data privacy laws. The source data underlying Figs 3a-b, 4a–f, 5a-e, and 6 are provided as a Source Data file. A minimum dataset for the interpretation, verification and extension of the research can be accessed at https://doi.org/10.5281/zenodo.5070592

Field-specific reporting

Please select the one below that is the best fit for your research. If you are not sure, read the appropriate sections before making your selection.

☒ Life sciences ☐ Behavioural & social sciences ☐ Ecological, evolutionary & environmental sciences

For a reference copy of the document with all sections, see nature.com/documents/nr-reporting-summary-flat.pdf

Life sciences study design

All studies must disclose on these points even when the disclosure is negative.

Sample size	No sample-size calculations were performed. Given that our morgue-based validation is performed on human remains, obtaining very high sample sizes is, naturally, limited. Nonetheless, we have strived to obtain the highest possible sample size; and we have increased our sample size compared with our previously published study (Sci. Adv. 6, eaba4243 (2020), doi:10.1126/sciadv.aba4243). We therefore believe that our sample size is sufficiently large to substantiate the feasibility of our method.
Data exclusions	No data were excluded.
Replication	In both experimental settings we performed at least 4 replications. All attempts at replication were succesful.
Randomization	Allocation was not randomized as validation of our method only requires a single experimental group.
Blinding	Blinding was not performed as our study does not rely on human assessment.

Reporting for specific materials, systems and methods

We require information from authors about some types of materials, experimental systems and methods used in many studies. Here, indicate whether each material, system or method listed is relevant to your study. If you are not sure if a list item applies to your research, read the appropriate section before selecting a response.

Materials & experimental systems

n/a	Involved in the study	
☒	☐	Antibodies
☒	☐	Eukaryotic cell lines
☒	☐	Palaeontology and archaeology
☒	☐	Animals and other organisms
☐	☒	Human research participants
☒	☐	Clinical data
☒	☐	Dual use research of concern

Methods

n/a	Involved in the study	
☒	☐	ChIP-seq
☒	☐	Flow cytometry
☒	☐	MRI-based neuroimaging

Human research participants

Policy information about studies involving human research participants

Population characteristics	Recently deceased human subjects. Age: 68 years to 87 years. Gender: three male, three female. Field validation study: two males and two females (aged between 34 and 73 years.)
Recruitment	Human cadavers were obtained through the body donation program (BDP) of the Department of Medical Biology, Section Clinical Anatomy and Embryology, of the Amsterdam University Medical Centers (UMC), location Academic Medical Center (AMC), in the Netherlands. A potential selection bias is present in the age distribution (favouring older age groups) of the participants in the lab validation, due to the nature of the body donation program. However, this bias is unlikely to influence results as the thermal properties are not altered. Moreover, there was no age selection bias in the field validation and the achieved PMI reconstruction accuracies of both validation studies are in close agreement.
Ethics oversight	The donation of these bodies to science occurred in accordance with Dutch legislation and the regulations of the medical ethical committee of the Amsterdam UMC, location AMC.

Note that full information on the approval of the study protocol must also be provided in the manuscript.
